# Antibacterial and Antibiofilm Effect of Cell-Free Supernatant of *Lactobacillus brevis* KCCM 202399 Isolated from Korean Fermented Food against *Streptococcus mutans* KCTC 5458

**DOI:** 10.4014/jmb.2109.09045

**Published:** 2021-10-17

**Authors:** Jong Ha Kim, Hye Ji Jang, Na-Kyoung Lee, Hyun-Dong Paik

**Affiliations:** Department of Food Science and Biotechnology of Animal Resource Konkuk University, Seoul 05029, Republic of Korea

**Keywords:** Probiotics, antibacterial effect, antibiofilm effect, *Streptococcus mutans*

## Abstract

This study aims to determine the antibiofilm effect of cell-free supernatant (CFS) of *Lactobacillus brevis* strains against *Streptococcus mutans* strains. To study the antibiofilm mechanism against *S. mutans* strains, antibacterial effects, cell surface properties (auto-aggregation and cell surface hydrophobicity), exopolysaccharide (EPS) production, and morphological changes were examined. The antibiofilm effect of *L. brevis* KCCM 202399 CFS as morphological changes were evaluated by scanning electron microscopy (SEM) and confocal laser scanning microscopy (CLSM), compared with the control treatment. Among the *L. brevis* strains, *L. brevis* KCCM 202399 showed the highest antibiofilm effect on *S. mutans* KCTC 5458. The antibacterial effect of *L. brevis* KCCM 202399 against *S. mutans* KCTC 5458 was investigated using the deferred method (16.00 mm). The minimum inhibitory concentration of *L. brevis* KCCM 202399 against *S. mutans* KCTC 5458 was 25.00%. Compared with the control treatment, *L. brevis* KCCM 202399 CFS inhibited the bacterial adhesion of *S. mutans* KCTC 5458 by decreasing auto-aggregation, cell surface hydrophobicity, and EPS production (45.91%, 40.51%, and 67.44%, respectively). *L. brevis* KCCM 202399 CFS inhibited and eradicated the *S. mutans* KCTC 5458 biofilm. Therefore, these results suggest that *L. brevis* KCCM 202399 CFS may be used to develop oral health in the probiotic industry.

## Introduction

*Streptococcus mutans*, a major microorganism of human dental caries in the oral cavity, can form biofilms on the teeth [1]. Their aggregation ability allows the colonization of *S. mutans* on the surface of human teeth, as a first step in the development of biofilms [2]. Thereafter, *S. mutans* glucosyltransferases (*gtfs*) begin to synthesize glucan, which increases the adherence of bacteria. In the presence of sucrose, *gtfs* also produce extracellular polysaccharides (EPSs) from sucrose [3]. Among the EPSs, glucan mediates the initial stage of adherence of oral bacteria on the tooth surface and stimulates the biofilm [4]. Once biofilm forms on a tooth surface, not only is it difficult to remove but also infects other tooth surfaces [5].

Dental caries, caused by *S. mutans*, is an oral disease affecting the health of adults and children [3]. According to the World Health Organization (WHO) report, more than 530 million children globally have dental caries in their primary teeth [6]. At present, dental caries has been treated using mechanical removal, such as tooth brushing and treatment with mouthwashes containing chlorhexidine and fluoride [7]. However, these methods can also destroy the ecological balance of the oral cavity or damage tissues [8].

Probiotics are live bacteria that can modulate the intestinal microflora when ingested in adequate amounts by the host [9, 10]. The characteristic properties of probiotics can prevent invasion and cellular adhesion of pathogenic bacteria [11]. In recent decades, the use of probiotics to prevent oral infections has significantly increased. Some of the specific *Lactobacillus* strains have shown the potential to interfere with oral ecology by inhibiting pathogenic bacteria such as *S. mutans* [12, 13]. Therefore, this study aims to identify antibacterial and antibiofilm effects of *Lactobacillus brevis* strains isolated from Korean fermented foods against oral pathogenic *S. mutans* strains.

## Materials and Methods

### Bacterial Strains and Growth Conditions

*L. brevis* strains were isolated using lactobacilli MRS (BD Bioscience, USA). *Lactobacillus rhamnosus* GG (Cell Biotech, Ltd., Korea) was used as a commercial control strain and obtained from the Korean Collection for Type Cultures (Korea). All *Lactobacillus* strains were cultured in MRS broth at 37°C for 24 h.

*S. mutans* KCTC 5124, KCTC 5458, and KCTC 5316 were obtained from the Korean Collection for Type Cultures. The strains were cultured in brain heart infusion broth (BHI, BD Bioscience, USA) supplemented with 3% sucrose at 37°C for 24 h and used as oral pathogenic bacteria.

### Preparation Cell-Free Supernatant (CFS) of *Lactobacillus* Strains

Lactobacillus strains were cultured in MRS broth at 37°C for 24 h. The CFSs of the strains CFS were centrifuged (12,000 ×*g* for 10 min at 4°C) and filtered through a 0.45-μm pore size syringe filter (Advantec, Japan) (pH 4.3) and stored at −80°C until further use.

### Antibacterial Effect of *L. brevis* Strains against *S. mutans* Strains

The antibacterial effect of *L. brevis* strains against *S. mutans* strains was investigated using the deferred method with minor modifications [14]. *L. brevis* strains (3 μl; 1 × 10^9^ CFU/mL) were spotted onto MRS agar and incubated at 37°C for 24 h. *S. mutans* strains (100 μl), as an indicator of oral diseases (1 × 10^7^ CFU/ml), were inoculated into 4 ml of BHI soft agar containing 3% sucrose, followed by soft agar overlay. The plate was incubated at 37°C for 24 h in an anaerobic incubator. Clear zones were measured and expressed in terms of millimeters.

### Minimum Inhibitory Concentration (MIC) of *L. brevis* Strains against *S. mutans* Strains

The MIC of *L. brevis* strains against *S. mutans* strains was investigated using the method described by Lim *et al*. [4] with some modifications. CFSs of the *L. brevis* strains were serially diluted two-fold using BHI broth containing 3% sucrose ranging from 100% to 0.78% in a 96 well plate (SPL, Korea). *S. mutans* strains (1 × 10^7^ CFU/ml) and diluted CFSs were added to each well. The lowest sample concentration that inhibited 99% of the inoculum was considered the MIC.

### Cell Surface Properties

The auto-aggregation and cell surface hydrophobicity of *S. mutans* KCTC 5458 were evaluated using the method of Sorroche *et al*. [15] with minor modifications. Five milliliters of *S. mutans* KCTC 5458 (1 × 10^7^ CFU/ml) was mixed with 5 ml of CFS diluted to 1/2 × MIC in BHI broth and incubated at 37°C for 24 h under anaerobic conditions; non-treated cells were used as a control. After incubation, cells were centrifuged (12,000 ×*g* for 10 min at 4°C), and the pellet was washed twice and resuspended with phosphate-buffered saline (PBS, pH 7.4; Hycolone, USA) to an optical density (OD) of 0.5 ± 0.05 at 600 nm (OD_initial_). The suspensions were incubated at 37°C for 24 h. Thereafter, the OD at 600 nm was measured for suspension (OD_Treatment_). Auto-aggregation (%) was calculated as follows:

$$\text{Auto-aggregation (\%) =}\left(\text{1-}\frac{{\text{OD}}_{\text{Treatment}}}{{\text{OD}}_{\text{Initial}}}\right)\text{×100}$$

After washing the cells, the absorbance at 600 nm (OD_Initial_) was adjusted to 0.5 ± 0. Chloroform (0.5 ml) was added to each cell suspension (2 ml) and pre-incubated for 10 min at 37°C. Thereafter, the mixtures were vortexed for 2 min and incubated for 15 min at 37°C. The aqueous phase was measured at 600 nm (OD_Treatment_). The cell surface hydrophobicity was calculated using the following formula:

$$\text{Cell surface hydrophobicity (\%) =}\left(\text{1-}\frac{{\text{OD}}_{\text{Treatment}}}{{\text{OD}}_{\text{Initial}}}\right)\text{×100}$$

### Analysis of Total EPS Production Rate

EPS production by *S. mutans* KCTC 5458 was measured by the phenol-sulfuric acid method with some modifications [16]. Five milliliters of *S. mutans* KCTC 5458 diluted to 10^7^ CFU/ml in BHI broth containing 3%sucrose was mixed with 5 ml of CFS diluted to 1/2 × MIC in BHI broth containing 3% sucrose and incubated at 37°C for 24 h under anaerobic conditions; non-treated cells were used as a control. After incubation, the treated mixtures were centrifuged at 12,000 ×*g* at 4°C for 10 min, and 1 ml of supernatant was mixed with 2 ml of 99% ethyl alcohol and incubated for 24 h at 4°C. After incubation, the mixture was centrifuged at 14,240 ×*g* for 15 min, and the pellets were resuspended in 500 μl of distilled water. In the cell suspension (100 μl), 5% phenol (100 μl), and 95% sulfuric acid (4 ml) were mixed; the mixture was vortexed, and incubated at 30°C for 10 min. The absorbance was calculated using the following formula:

$$\text{EPS production rate (\%) =}\frac{{\text{OD}}_{\text{Treatment}}}{{\text{OD}}_{\text{Control}}}\text{×100}$$

### Biofilm Assay

Biofilm inhibition and eradication were measured using a crystal violet assay, with some modifications [17]. Overnight cultured *S. mutans* KCTC 5458 was diluted to 10^7^ CFU/ml in BHI broth containing 3% sucrose. To determine the inhibitory effect of *L. brevis* CFS on the formation of *S. mutans* biofilm, 50 μl of bacterial cultures and 50 μl of CFS diluted to 1/2 × MIC in BHI broth containing 3% sucrose were transferred to a 96 well plate and incubated at 37°C for 24 h; untreated cells were used as a control. After biofilm formation, the cell suspensions were removed using a micropipette. The plates were washed twice with 150 μl of PBS. Plates were dried at 37°C for 20 min. Thereafter, 1% crystal violet was added to each well to stain the biofilm-forming cells for 30 min at room temperature. After dyeing, the plate was rinsed and dissolved in a solution of 30% methanol and 10% acetic acid. The OD of each sample was measured at 570 nm using a microplate reader (Molecular Devices, USA).

To investigate the effect of eradication on the formation of *S. mutans* biofilms, the cell density was adjusted to 10^7^ CFU/ml in BHI broth containing 3% sucrose, and 100 μl of cell suspension was inoculated into 96 well plate and incubated at 37°C for 24 h under anaerobic conditions; non-treated cells were used as a control. After incubation, each well was washed twice with 150 μl PBS. One hundred microliters of *L. brevis* CFS (1/2 × MIC and MIC) was added to each well and incubated at 37°C for 24 h. Non-treated cells were used as a control. The results were quantified as follows.

$$\text{Biofilm inhibition and eradication rate (\%) =}\left(\text{1-}\frac{{\text{OD}}_{\text{Treatment}}}{{\text{OD}}_{\text{Initial}}}\right)\text{×100}$$

### Scanning Electron Microscopy (SEM) Analysis

SEM was performed to investigate the biofilm inhibition effect of *L. brevis* KCCM 202399 CFS on *S. mutans* KCTC 5458 biofilm using a modified method [18]. Overnight cultured *S. mutans* KCTC 5458 was diluted to 10^7^ CFU/ml using BHI broth containing 3% sucrose. Two milliliters of bacterial suspension and 2 ml of CFS diluted to 1/2 × MIC in BHI broth containing 3% sucrose were cultured in each well of a six-well plate containing glass coupons and incubated at 37°C for 24 h under anaerobic conditions. The control group was treated with BHI broth containing 3% sucrose. The biofilms formed on the glass coupons were fixed with 2.5% glutaraldehyde in PBS at 4°C for 1 h. The fixed samples were washed twice with PBS and dehydrated for 30 min using gradually increasing concentrations of ethanol solutions (50%, 70%, 80%, 90%, and 100%). Ethanol was replaced with isoamyl acetate, and the coupons were dried in a freeze dryer and then coated with platinum particles (15 mV for 1.5 min). The *S. mutans* KCTC 5458 biofilm was observed using a field-emission scanning electron microscope (FESEM; SU8010; Hitachi High-Technologies Co., Japan).

### Confocal Laser Scanning Microscopy (CLSM) Analysis

CLSM was performed to evaluate the biofilm inhibition effect of *L. brevis* KCCM 202399 CFS on *S. mutans* KCTC 5458 biofilm. Biofilms of *S. mutans* KCTC 5458 were prepared using the same protocol as described in section 2.8. After biofilm formation, the glass coupons were washed twice with PBS. Live and dead cells were stained with 1 μM SYTO9 and propidium iodide (PI) for 20 min in the dark at room temperature. After staining, the glass coupons were washed twice with PBS and observed under a Zeiss LSM 800 microscope (Carl Zeiss, Germany) using a 10 × objective lens and an appropriate standard filter.

### Statistical Analysis

All experiments were repeated three times with duplicate samples, and the results are presented as the mean ± standard deviation. All statistical analyses were performed using SPSS 18.0. Significant differences among means were determined using one-way analysis of variance (ANOVA).

## Results

### Antibacterial Effect against *S. mutans* Strains

The antibacterial effects of *Lactobacillus* strains against *S. mutans* strains are presented in Table 1. Among *S. mutans* strains, *L. brevis* strains showed a higher antibacterial effect against *S. mutans* KCTC 5458 than against *S. mutans* KCTC 5124 and *S. mutans* KCTC 5316, except for *L. rhamnosus* GG. Antibacterial effects of *L. rhamnosus* GG were 7.55 ± 1.3 mm and 9.11 ± 1.0 mm against *S. mutans* KCTC 5124 and *S. mutans* KCTC 5316, respectively (Table 1; *p* < 0.05). *L. brevis* strains showed a high antibacterial effect against *S. mutans* KCTC 5458 (all *Lactobacillus* strains examined had an inhibition zone over 10 mm). *L. rhamnosus* GG and *L. brevis* KCCM 202399 showed higher antibacterial effect against *S. mutans* KCTC 5458 than other *L. brevis* strains (16.66 ± 1.0 mm and 16.00 ± 1.0 mm, respectively; Table 1). Table 2 shows the MIC of *L. brevis* strains CFS against *S. mutans* strains. *L. brevis* strains CFS showed antibacterial effects against *S. mutans* KCTC 5458, except for *S. mutans* KCTC 5124 and *S. mutans* KCTC 5316. The MICs of *L. rhamnosus* GG were 25%, 6.25%, and 25% against *S. mutans* KCTC 5124, *S. mutans* KCTC 5458, and *S. mutans* KCTC 5316, respectively, while those of *L. brevis* KCCM 202399 were 25%, 6.25%, and 25%, respectively. *L. brevis* KCCM 202399 CFS showed better antibacterial effects against *S. mutans* strains than the other *L. brevis* strains.

### Cell Surface Properties

The effects of *L. brevis* strains CFS on auto-aggregation and cell surface hydrophobicity of *S. mutans* KCTC 5458 are shown in Table 3. Treatment with *L. brevis* strains CFS decreased auto-aggregation (*p* < 0.05) and cell-surface hydrophobicity (*p* < 0.05) of *S. mutans* KCTC 5458, compared with the negative control. The auto-aggregation ability of *S. mutans* KCTC 5458 treated with *L. brevis* KCCM 202399 and *L. brevis* KU15147 decreased by 45.91%and 49.11%, respectively. Additionally, *S. mutans* KCTC 5458 treated with *L. rhamnosus* GG was 46.35%. The cell surface hydrophobicity of *S. mutans* KCTC 5458 treated with *L. rhamnosus* GG CFS and *L. brevis* KCCM 202399 CFS was 32.97% and 40.51%, respectively. Our results showed that *Lactobacillus* strains CFS inhibited bacterial adhesion by decreasing the auto-aggregation and cell-surface hydrophobicity of *S. mutans* KCTC 5458.

### EPS Production Rate

**Fig. 1:**
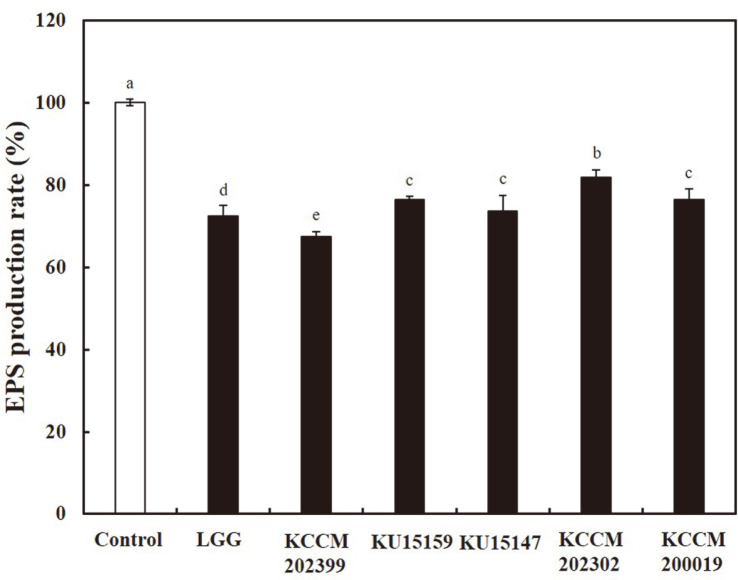
EPS production rate of *Streptococcus mutans* KCTC 5458 treated with cell-free supernatant (CFS) of *Lactobacillus brevis* strains. □, Control (treated *L. brevis* strains); ■, *L. brevis* strains; LGG, *L. rhamnosus* GG. Each value represents the mean ± standard deviation, with ^a-d^different letters on each bar representing significant differences (*p* < 0.05).

The EPS production rate of *S. mutans* KCTC 5458 treated with *L. brevis* strains CFS was evaluated using a modified phenol-sulfuric acid method [16]. Fig. 1 presents the inhibitory effect of *L. brevis* strain CFS on EPS production rate (*p* < 0.05). Treatment with *L. brevis* KCCM 202399 CFS resulted in the lowest EPS production rate (67.44%). For treatment with *L. rhamnosus* GG CFS, the EPS production rate was 72.35%, followed by treatment with *L. brevis* KU15147, and *L. brevis* KCCM 200019 (73.66% and 76.44%, respectively). As shown in Fig. 1 and Supplementary Tables 1, 2, and 3, *L. brevis* KCCM 202399, *L. brevis* KU15159, and *L. brevis* KU15147 CFS had a greater inhibitory effect on *S. mutans* growth and EPS production than other *L. brevis* strains.

### Biofilm Inhibition and Eradication Effects of CFS

**Fig. 2:**
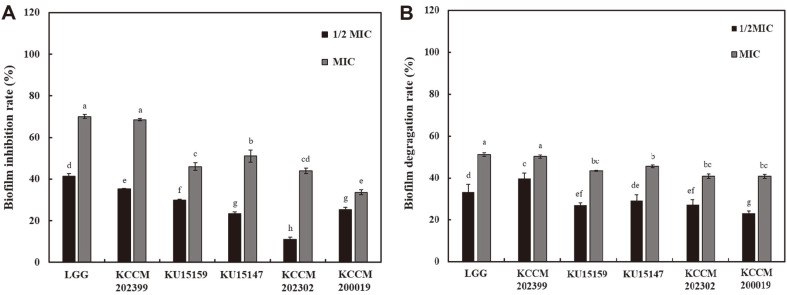
Antibiofilm effects of cell-free supernatant (CFS) of *Lactobacillus brevis* strains on *Streptococcus mutans* KCTC 5458. (**A**), Biofilm inhibition effect of CFS; (**B**), Biofilm eradication effect of CFS. LGG, *L. rhamnosus* GG; KCCM 202399, *L. brevis* KCCM 202399; KU15159, *L. brevis* KU15159; KU15147, *L. brevis* KU15147; KCCM 202302, *L. brevis* KCCM 202302; KCCM 200019, *L. brevis* KCCM 200019. Each value represents the mean ± standard deviation, with ^a-g^different letters on each bar representing significant differences (*p* < 0.05).

The inhibitory effect of *L. brevis* CFS on *S. mutans* KCTC 5458 biofilm is shown in Fig. 2A (*p* < 0.05); the concentration of CFS ranging from 1/2 × MIC and MIC inhibited the formation of *S. mutans* biofilm. Upon treatment with CFS at MIC concentration, *L. brevis* KCCM 202399 showed highest inhibition effects against *S. mutans* (68.54%). In addition, the inhibition effect of *L. rhamnosus* GG CFS was 70.05%. This inhibitory tendency was similarly observed at 1/2 MIC concentrations. Upon treatment with CFS at 1/2 MIC concentrations, the inhibition effects of *L. rhamnosus* GG CFS and *L. brevis* KCCM 202399 CFS were 41.45% and 35.28%, respectively. The degradation effect of *L. brevis* CFS on mature biofilms is shown in Fig. 2B (*p* < 0.05). Upon treatment with CFS MIC, the degradation effect of *L. rhamnosus* GG CFS, *L. brevis* KCCM 202399 CFS, and *L. brevis* KU15147 CFS was 51.19%, 50.29%, and 45.44%, respectively. However, upon treatment with CFS at 1/2 MIC concentrations, *L. brevis* KCCM 202399 CFS showed a greater degradation effect than the other *L. brevis* strains CFS (39.75%; *p* < 0.05). The biofilm inhibition and degradation effects of *L. brevis* CFS were dose-dependent. As shown in Fig. 2, *L. brevis* KCCM 202399 CFS showed greater biofilm inhibition and eradication effects against *S. mutans* than other *L. brevis* strains CFS (*p* < 0.05).

### SEM Analysis on Glass Coupon

**Fig. 3:**
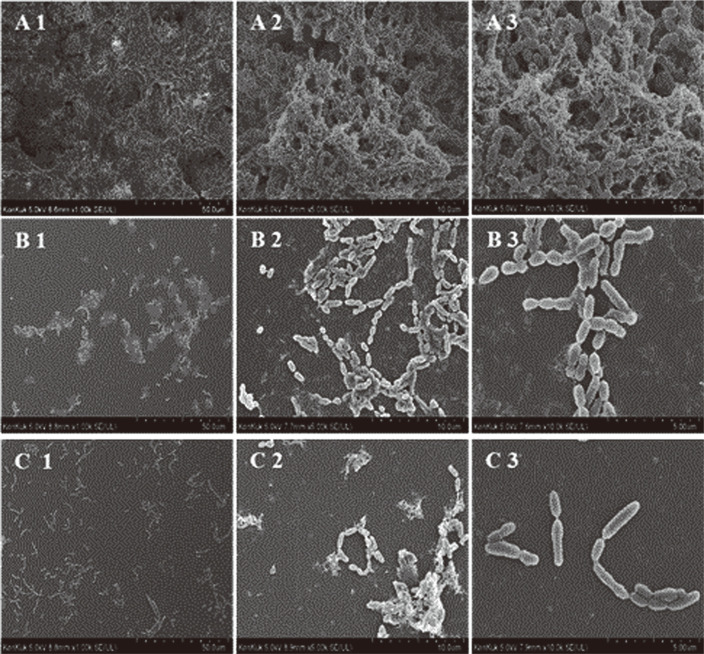
*Streptococcus mutans* KCTC 5458 biofilm on glass coupons surface treatment with cell-free supernatant (CFS) of *Lactobacillus brevis* KCCM 202399 visualized by scanning electron microscopy (SEM) images (magnification: × 1,000, × 5,000, and × 10,000). A group: control group (untreated with *L. brevis* CFS); B group: treated with *L. rhamnosus* GG CFS; C group: treated with *L. brevis* KCCM 202399 CFS.

The effects of *L. brevis* KCCM 202399 CFS on biofilm formation by *S. mutans* KCTC 5458 on glass coupons were also evaluated by SEM (Fig. 3). In the control group, *S. mutans* KCTC 5458 formed numerous bacterial cells and a large biofilm on the glass coupons (Fig. 3A). However, biofilm structures of *S. mutans* KCTC 5458 treated-*L. rhamnosus* GG CFS and *L. brevis* KCCM 202399 CFS were spread, resulting in decreased bacterial cells and biofilms on glass coupons (Figs. 3B and 3C). Considering the results of the other experiments, including cell surface properties (Table 3), EPS production (Fig. 1), biofilm inhibition and degradation effects (Fig. 2), the inhibitory effect of probiotic *L. brevis* KCCM 202399 CFS from *S. mutans* biofilm was confirmed.

### CLSM Analysis on Glass Coupon

**Fig. 4:**
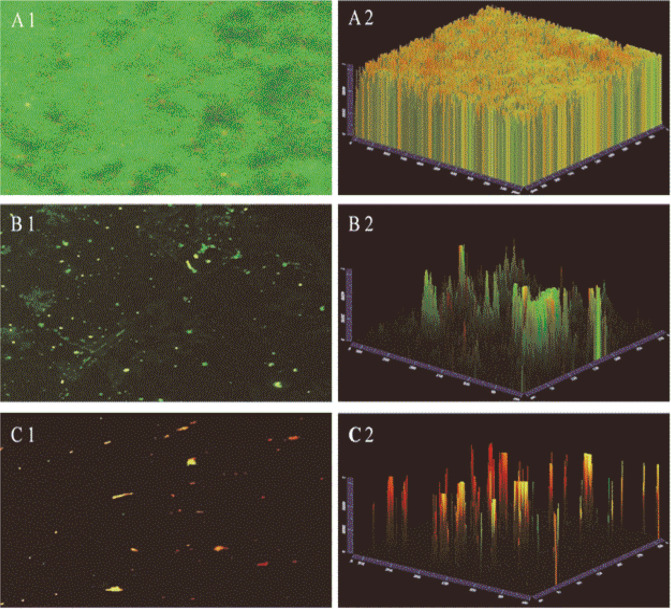
Confocal laser scanning microscopy (CLSM) images of *Streptococcus mutans* KCTC 5458 biofilm on glass coupons surface treated with *Lactobacillus brevis* KCCM 202399 cell-free supernatant (CFS) (× 100 magnification). A group: control group (untreated with *L. brevis* CFS); B group: treated with *L. rhamnosus* GG CFS; C group: treated with *L. brevis* KCCM 202399 CFS.

The antibiofilm and antibacterial effects of *L. brevis* KCCM 202399 CFS against *S. mutans* KCTC 5458 on glass coupons were observed via CLSM. CLSM images showed *S. mutans* biofilms with viable and non-viable cells (green and red, respectively). In the control, *S. mutans* KCTC 5458 showed a dense biofilm structure and biofilm cells (Fig. 4A1, 2). However, the biofilm structures of *L. rhamnosus* GG CFS- and *L. brevis* KCCM 202399 CFS-treated *S. mutans* KCTC 5458 were decreased. Furthermore, *L. brevis* KCCM 202399 CFS showed significantly reduced viability of *S. mutans* KCTC 5458 biofilm cells compared with *L. rhamnosus* GG CFS (Figs. 4B and 4C).

## Discussion

Dental caries is a major oral disease that is multi-species biofilm-mediated. Dental plaque, which is a multi-species biofilm, is transformed from cariogenic to non-cariogenic plaque. *S. mutans* is a cariogenic bacteria in dental plaque that colonizes the tooth surface and forms biofilms [5]. Once *S. mutans* forms a biofilm, it is difficult to remove; therefore, its early control is important. This study was aimed at investigating the antibacterial and antibiofilm effects of *L. brevis* strains isolated from kimchi.

In this study, methods were developed to screen the antibacterial effect of *L. brevis* strains against *S. mutans* strains. The results showed that *L. brevis* strains showed a higher antibacterial effect against *S. mutans* KCTC 5458 than against *S. mutans* KCTC 5124 and *S. mutans* KCTC 5316. Thereafter, antibacterial effect of *L. brevis* strains against *S. mutans* strains was investigated by using *L. brevis* CFS; it was confirmed that *L. brevis* strains have a higher antibacterial effect on *S. mutans* KCTC 5458 than on *S. mutans* KCTC 5124 and *S. mutans* KCTC 5316. Some *Lactobacilli* strains can metabolize sucrose, co-aggregate with *S. mutans*, and are often tolerant to fluoride [19]. Therefore, probiotic supernatant could be safely used as an antibacterial agent for treating dental plaque. Taku *et al*. [20] reported that the “expression” or “sensitivity” of *gtf* gene, which synthesizes water in-soluble or soluble glucans from sucrose, would be affected differently in different *S. mutans* strains. CFSs of all *L. brevis* strains showed greater antibacterial effect against *S. mutans* KCTC 5458 than against *S. mutans* KCTC 5124 and *S. mutans* KCTC 5316. In particular, *L. brevis* KCCM 202399 CFS showed an antibacterial effect at the lowest concentration among the *L. brevis* strains (Table 2). Therefore, we focused on the antibacterial effect of *L. brevis* CFS against *S. mutans* KCTC 5458.

Auto-aggregation, cell surface hydrophobicity, and EPS production changes in *S. mutans* are important to prevent *S. mutans* adhesion, colonization, and early biofilm formation [21]. Auto-aggregation of *S. mutans* is beneficial for its adhesion to tooth surface, as the resulting biofilm formed prevents this bacterium from an adverse external environment [22]. *S. mutans* has a high overall proportion of hydrophobic bacteria, and its cell surface hydrophobicity may play a role in the adherence of oral bacteria to the tooth surface [23]. Therefore, we investigated the effect of *L. brevis* CFS on the auto-aggregation ability and cell surface physiochemical properties of *S. mutans* KCTC 5458. In this study, *L. brevis* KCCM 202399 CFS showed the highest reduction in auto-aggregation and cell surface hydrophobicity of *S. mutans* KCTC 5458. In another study, auto-aggregation of *S. mutans* ATCC 25175 treated with *L. brevis* BBE-Y52 was higher than that in the presence of other *Lactobacillus* strains [24]. In addition, Bacillus velezensis K68-treated *L. brevis* strains exhibited increased cell surface hydrophobicity, compared with the untreated control [25]. EPS produced by *S. mutans* is a major factor in biofilm formation. As sucrose exists in oral conditions, *gtfs* from *S. mutans* plays critical roles in the development of virulent dental plaque [26]. In the presence of *L. brevis* CFS, EPS production by *S. mutans* KCTC 5458 decreased. In particular, *L. brevis* KCCM 202399 CFS showed the highest reduction in EPS production by *S. mutans*. These results suggest that *L. brevis* KCCM 202399 reduces sucrose-dependent EPS production by downregulating *gtfs*. In our previous study, *L. brevis* KU15153 CFS decreased EPS production by approximately 41% (*p* < 0.05) [27]. A previous study reported that biosurfactants produced by probiotics have antibacterial and anti-adhesive properties [28]. The biosurfactants in metabolites exuded by *Lactobacilli* interfere with the adhesion of cells. These decrease the hydrophobicity of the cell surface substratum and interfere with the progression of microbial adhesion ability [29]. In addition, Tahmourespour *et al*. [30] reported that *Lactobacillus acidophilus*-derived biosurfactant down-regulated *gtfs* B and C genes, and virulence factors were associated with glucan in dental plaque.

Changes in cell surface properties and EPS production by *L. brevis* KCCM 202399 CFS also affected biofilm formation by *S. mutans* KCTC 5458. In this study, 1/2 MIC and MIC concentrations of *L. brevis* CFS were used for treatment and the inhibition rate of *S. mutans* biofilm as a function of CFS concentration was investigated. The biofilm formed by *S. mutans* in the presence of *L. brevis* strains CFS exhibited a dose-dependent reduction in biomass compared to the control group that did not receive CFS. In particular, *L. brevis* KCCM 202399 CFS showed the highest inhibitory effect against *S. mutans* KCTC 5458 at 1/2 MIC and MIC levels. In our previous study, *L. rhamnosus* GG was reported to have a significant antibacterial effect against *S. mutans* [4]. Ahn *et al*. [31] reported that lipoteichoic acid of probiotics could inhibit biofilm formation by *S. mutans*. Additionally, bacteriocin, an antibacterial substance produced by probiotic *Lactobacilli*, can kill gram-positive bacteria by disrupting their cell membranes, inhibit their growth by lowering pH and hamper bacterial DNA synthesis by producing hydrogen peroxide [32]. We also conducted SEM and CLSM analyses to investigate the reduction in biofilm formation and viability of biofilm cells as imaging. *S. mutans* KCTC 5458 treated with *L. brevis* KCCM 202399 CFS showed that the biofilm was dispersed with little aggregation, and the number of cells on glass coupons was decreased compared with that for the control (Fig. 3). Compared with *L. rhamnosus* GG, *L. brevis* KCCM 202399 showed a higher number of dead biofilm cells, as evidenced by PI staining (Fig. 4). Generally, mature biofilms are more difficult to remove than early biofilms. Biofilms can protect oral bacteria, and *S. mutans* can induce membrane proteins to migrate and overcome cellular damage caused by environmental stress [5, 33]. The biofilm degradation effect is also important to examine the antibiofilm effect against *S. mutans*; however, eradication effect of probiotics on mature biofilms have rarely been reported. *L. brevis* KCCM 202399 CFS showed the highest biofilm eradication effect against *S. mutans* KCTC 5458 at 1/2 MIC and MIC levels, demonstrating its action through biofilm formation and disruption of mature biofilm.

Six *L. brevis* strains isolated from kimchi were tested for antibacterial effects against *S. mutans* strains. The results showed that *L. brevis* KCCM 202399 had the highest antibacterial and antibiofilm effects among the *L. brevis* strains. Furthermore, *L. brevis* KCCM 202399 showed more antibacterial effects against *S. mutans* KCTC 5458 than against *S. mutans* KCTC 5124 and *S. mutans* KCTC 5316. *L. brevis* KCCM 202399 CFS inhibited and eradicated the biofilm of *S. mutans* KCTC 5458 by decreasing its auto-aggregation, cell-surface hydrophobicity, and EPS production. The antibiofilm effects of CFS against *S. mutans* KCTC 5458 were also confirmed by SEM and CLSM. Therefore, this study suggests that *L. brevis* KCCM 202399 could be used as a functional food in the food industry.

## Figures and Tables

**Table 1:** Inhibition effect of *Lactobacillus* strains against *Streptococcus mutans* strains.

Oral pathogenic bacteria	Inhibitory diameter (mm)					

	LGG^1)^	KCCM 202399^2)^	KU15159^3)^	KU15147^4)^	KCCM 202302^5)^	KCCM 200019^6)^
*S. mutans* KCTC 5124	7.55 ± 1.33^a^	7.66 ± 0.86^a^	5.66 ± 0.86^b^	6.33 ± 1.22^a^	6.11 ± 1.16^b^	4.88 ± 1.05^b^
*S. mutans* KCTC 5458	16.27 ± 2.10^a^	16.11 ± 1.45^a^	13.33 ± 1.73^bc^	14.83 ± 0.93^ab^	11.72 ± 1.60^cd^	10.66 ± 1.32^d^
*S. mutans* KCTC 5316	9.44 ± 1.42^a^	8.56 ± 1.67^a^	6.89 ± 0.78^b^	6.33 ± 1.00^b^	6.78 ± 2.05^b^	5.33 ± 0.71^b^

^1-6)^LGG, *L. rhamnosus* GG; KCCM 202399, *L. brevis* KCCM 202399; KU15159, *L. brevis* KU15159; KU15147, *L. brevis* KU15147; KCCM 202302, *L. brevis* KCCM 202302; KCCM 200019, *L. brevis* KCCM 200019.

All values are mean ± standard deviation.

^a-d^Values with different letters in the same row are significantly different (*p* < 0.05). 1-6) LGG, *L. rhamnosus* GG; KCCM 202399, *L. brevis* KCCM 202399; KU15159, *L. brevis* KU15159; KU15147, *L. brevis* KU15147; KCCM 202302, *L. brevis* KCCM 202302; KCCM 200019, *L. brevis* KCCM 200019.

All values are mean ± standard deviation.

^a-d^Values with different letters in the same row are significantly different (*p* < 0.05).

**Table 2:** Antibacterial effect of the *Lactobacillus* strains cell free supernatant (CFS) against *S. mutans* strains.

Oral pathogenic bacteria	Minimal inhibitory concentration (%)					

	LGG^1)^	KCCM 202399^2)^	KU15159^3)^	KU15147^4)^	KCCM 202302^5)^	KCCM 200019^6)^
*S. mutans* KCTC 5124	25 ± 0.0^a^	25 ± 0.0^a^	50 ± 0.0^b^	50 ± 0.0^b^	50 ± 0.0^b^	50 ± 0.0^b^
*S*. *mutans* KCTC 5458	6.25 ± 0.0^a^	6.25 ± 0.0^a^	12.5 ± 0.0^b^	12.5 ± 0.0^b^	25 ± 0.0^c^	25 ± 0.0^c^
*S. mutans* KCTC 5316	25 ± 0.0^a^	25 ± 0.0^a^	50 ± 0.0^b^	50 ± 0.0^b^	50 ± 0.0^b^	50 ± 0.0^b^

^1-6)^LGG, *L. rhamnosus* GG; KCCM 202399, *L. brevis* KCCM 202399; KU15159, *L. brevis* KU15159; KU15147, *L. brevis* KU15147; KCCM 202302, *L. brevis* KCCM 202302; KCCM 200019, *L. brevis* KCCM 200019.

All values are mean ± standard deviation.

^a-c^Values with different letters in the same row are significantly different (*p* < 0.05).

**Table 3:** Effects of the cell-free supernatant of *Lactobacillus* strains on auto-aggregation and cell surface hydrophobicity of S.mutans KCTC 5458.

	Probiotics strains					

	LGG^1)^	KCCM 202399^2)^	KU15159^3)^	KU15147^4)^	KCCM 202302^5)^	KCCM 200019^6)^
Auto-aggregation (%)						
Control^7)^	67.34 ± 3.3	67.34 ± 3.3	67.34 ± 3.3	67.34 ± 3.3	67.34 ± 3.3	67.34 ± 3.3
Treated^8)^	46.35 ± 3.18^a^	45.91 ± 1.97^a^	56.94 ± 3.21^b^	49.11 ± 0.88^c^	59.76 ± 2.26^d^	54.54 ± 1.7^c^
Hydrophobicity (%)						
Control	56.39 ± 3.5	56.39 ± 3.5	56.39 ± 3.5	56.39 ± 3.5	56.39 ± 3.5	56.39 ± 3.5
Treated	32.97 ± 0.39^a^	40.51 ± 1.04^b^	48.44 ± 4.43^c^	45.18 ± 1.72^c^	52.19 ± 3.67^d^	45.93 ± 7.61^c^

^1-8)^LGG, *L. rhamnosus* GG; KCCM 202399, *L. brevis* KCCM 202399; KU15159, *L. brevis* KU15159; KU15147, *L. brevis* KU15147; KCCM 202302, *L. brevis* KCCM 202302; KCCM 200019, *L. brevis* KCCM 200019; control, treated with probiotic CFS.

^a-c^Values with different letters in the same row are significantly different (*p* < 0.05).

All values are mean ± standard deviation.
